# Antidepressant Effects of Rosemary Extracts Associate With Anti-inflammatory Effect and Rebalance of Gut Microbiota

**DOI:** 10.3389/fphar.2018.01126

**Published:** 2018-10-02

**Authors:** Ying Guo, Jianping Xie, Xia Li, Yun Yuan, Lanchun Zhang, Weiyan Hu, Haiyun Luo, Haofei Yu, Rongping Zhang

**Affiliations:** ^1^School of Pharmaceutical Science & Yunnan Key Laboratory of Pharmacology for Natural Products, Kunming Medical University, Kunming, China; ^2^School of Basic Medical Sciences, Kunming Medical University, Kunming, China; ^3^Library, Yunnan Minzu University, Kunming, China; ^4^Department of Zoology, Kunming Medical University, Kunming, China

**Keywords:** depression, hippocampus, inflammation, gut microbiota, microglia, chronic restraint stress, rosemary extract

## Abstract

It is currently believed that inflammation acts as a central part in the pathophysiology of depression. Rosemary extracts (RE), the crucial active constituents extracted from *Rosmarinus officinalis* Linn, have drawn wide concerns because of their potential for anti-inflammatory effects. However, no study has highlighted the antidepressant effects of RE on chronic restraint stress (CRS) mice, and the inflammatory mechanisms related to gut microbiome have not yet been elucidated. This study showed that depressive-like behaviors, gut microbiota dysbiosis, and activation of inflammatory reactions in the hippocampus and serum of CRS mice, as well as activation of inflammatory reactions in BV-2 microglia cells induced by lipopolysaccharide (LPS), could be attenuated by RE. We found that the pretreatment with RE increased the time in the center of open field test (OFT), and decreased immobility duration in tail suspension test (TST) as well as forced swimming test (FST). Furthermore, RE enhanced the sequences proportion of Lactobacillus and Firmicutes, and reduced the sequences proportion of Bacteroidetes and Proteobacteria in feces. Moreover, RE obviously suppressed protein expression of IL-1β, TNF-α, p-NF-κ B p65 and Iba1 in hippocampus, and elevated BDNF as well as p-AKT/AKT expression. Importantly, pre-incubation with RE protected microglia by alleviating protein expression of IL-1β, TNF-α and p-NF-κ B p65 induced by LPS. Additionally, RE downregulated the level of IL-1β and TNF-α in serum. In conclusion, this study showed the antidepressant effects of RE are mediated by anti-inflammatory effects in hippocampus, serum and BV-2 microglia as well as rebalancing gut microbiota.

## Introduction

Major depressive disorder (MDD) is a severe mental sickness (Lemogne et al., 2013) featured by loss of interest, disturbed sleep, lack of energy, and suicidality, which gives rise to the highest ratio of worldwide burden according to mental disorders (Hou et al., 2016). MDD has been strongly associated with gut microbiome diversity (Winter et al., 2018) and gut microbiota composition (Zheng et al., 2016). Previous research has established *Lactobacillus helveticus* NS8 plays an anti-depressant role in rats subjected to CRS depression and this effect is rooted from the microbiota-gut-brain axis (Liang et al., 2015). Depression affects serum cytokines alterations, including interleukin-1β (IL-1β), IL-6, and IL-8 levels, and leads to inflammatory processes (Kim et al., 2018). Additionally, depression caused by CRS may be related to inflammatory pathways, including tumor necrosis factor (TNF) signaling pathway, nuclear factor κB (NF-κB) signaling pathway and Toll-like receptor signaling pathway (Wang et al., 2017), which indicates inflammatory and microglial activation are involved in depressive-like behaviors (Yamawaki et al., 2018). The mRNA and protein expression levels of brain-derived neurotrophic factor (BDNF) are remarkably lessened in hippocampus in the rats exposed to CRS (Zhang et al., 2017). Further, CRS can induce cognitive impairment and hippocampal neuronal apoptosis in mice, associated with the downregulation of AKT signaling pathway (Huang et al., 2015).

Despite the consequences following depression are serious, effective antidepressant drugs are limited. Rat experiments have demonstrated that RE owned effective anti-inflammatory function (de Almeida Goncalves et al., 2018). However, no study has highlighted the antidepressant effects of RE on CRS mice, and the inflammatory mechanisms related to gut microbiome have not yet been elucidated.

Thus, in this study, we investigated the effects of RE on gut microbiota, inflammation, as well as behaviors of CRS mice to focus on inflammation and unbalance of gut microbiota as pathological mechanisms and as potential therapeutic targets of depression.

## Materials and Methods

### Reagents

Rosemary extracts (Kunming Pharmaceutical Co., China.) are the crucial active constituents extracted from *Rosmarinus officinalis* Linn, containing 60% carnosic acid. RE were dissolved in 1% Tween-80 to prepare a suspension with the concentration of 100 mg/mL. Rabbit anti-BDNF, rabbit anti-Iba1 and rabbit anti-IL-1β were purchased from Abcam (Shanghai, China). Rabbit anti-TNF-α, rabbit anti-p-NF κ B p65, rabbit anti-NF κ B p65, rabbit anti-AKT, rabbit anti-p-AKT 473, β-actin and horseradish peroxidase conjugated anti-rabbit IgG were acquired from Cell Signaling Technology (Boston, MA, United States). Enzyme-linked immunosorbent assay (ELISA) kits were obtained from RD, Bio-Techne (Emeryville, CA, United States). E.Z.N.A. stool DNA Kit was purchased from Omega Bio-tek (Norcross, GA, United States), AxyPrep DNA Gel Extraction Kit was acquired from Axygen Biosciences (Union City, CA, United States).

### Animals and Treatment

Adult male ICR mice were obtained from Kunming Medical University. Animals were housed in groups of five per cage under a normal light cycle room and were allowed to adapt to their new housing conditions for 1 week before the onset of the experiment. All animals had free access to food and water. All animal handling and surgical procedures were approved by the Animal Research Ethics Committee of Kunming Medical University.

The mice were randomly assigned to groups of CON, CRS and RE + CRS, and were given with 1% Tween-80 (10 mL/kg), 1% Tween-80 (10 mL/kg) and RE (100 mg/kg), respectively by gavage for 21 days. Mice of the last two groups were exposed to restraint stress for 21 days. Every day, mice were placed in a horizontal resting position inside a well-ventilated (12 holes, 0.5 mm diameter) 50 mL tube and after 4 h they were unrestrained.

### Behavioral Test

#### OFT

Mice were gently placed in an open field, a box (50 cm × 50 cm × 28 cm) with its floor divided into 25 squares. Nine squares were defined as the center and the 16 squares along the walls as the periphery. Movements were digitally captured and analyzed in a 6 min trial by Smart video tracking software V3.0 (Panlab, Spain).

#### TST

Mice were suspended upside down by tail 40 cm above the floor by adhesive tape placed 1 cm from the tail tip. During a 6 min test period, the immobility time was scored for the last 4 min by the Smart video tracking software V3.0.

#### FST

Mice were placed in a vertical transparent cylinder (30 cm in height and 12 cm in diameter) containing tap water at 25 ± 1°C and 20 cm in depth. During a 6 min test period, the immobility time of mice was recorded for the last 4 min by the Smart video tracking software V3.0.

### Analysis of Intestinal Microflora Community Diversity

DNA Extraction and PCR Amplification: Microbial DNA was extracted from feces samples from CON, CRS, RE + CRS groups using the E.Z.N.A. stool DNA Kit according to the manufacturer’s protocols. The V1-V3 hypervariable regions of the bacteria 16S rRNA gene were amplified with primers 27F (5′- AGAGTTTGATCCTGGCTCAG -3′) and 533R (5′- TTACCGCGGCTGCTGGCAC -3′) by thermocycler PCR system (GeneAmp 9700, ABI, United States). The PCR reactions were conducted using the following program: 3 min of denaturation at 95°C, 30 cycles of 30 s at 95°C, 30 s for annealing at 55°C, and 45 s for elongation at 72°C, and a final extension at 72°C for 10 min. PCR reactions were performed in triplicate 20 μL mixture containing 4 μL of 5 × FastPfu buffer, 2 μL of 2.5 mM dNTPs, 0.8 μL of each primer (5 μM), 0.4 μL of FastPfu polymerase and 10 ng of template DNA.

Illumina MiSeq Sequencing: Amplicons were extracted from 2% agarose gels and purified using the AxyPrep DNA gel extraction kit according to the manufacturer’s instructions, and quantified using QuantiFluor-ST (Promega, United States). The purified amplicons were pooled in equimolar quantities and paired-end sequenced (2 × 300) on an Illumina MiSeq platform according to standard protocols.

Processing of Sequencing Data: Raw fastq files were demultiplexed, quality-filtered by Trimmomatic and merged by FLASH with the following criteria: (i) The reads were truncated at any site receiving an average quality score <20 over a 50-bp sliding window. (ii) Primers were exactly matched allowing maximum 2 nucleotide mismatching, and reads containing ambiguous bases were removed. (iii) Sequences whose overlap longer than 10 bp were merged according to their overlap sequence. Operational taxonomic units (OTUs) were clustered with 97% similarity cutoff using UPARSE (version 7.1^1^) and chimeric sequences were identified and removed using UCHIME. The taxonomy of each 16S rRNA gene sequence was analyzed by RDP Classifier algorithm^2^ against the Silva (SSU123) 16S rRNA database using confidence threshold of 70%. The data were analyzed on the free online platform of Majorbio I-Sanger Cloud Platform^3^.

### BV-2 Microglia Cell Culture and Treatment

BV-2 microglia cells were cultured in Dulbecco’s modified Eagle’s medium (DMEM), supplemented with 10% fetal calf serum at 37°C in a humidified incubator under 5% CO_2_. The cells were divided into CON, LPS induced and RE + LPS. The cells were pretreated with phosphate buffer saline (PBS), PBS, RE (5 μM, 5 μM was the amount of substance of carnosic acid, and RE contained 60% carnosic acid.), respectively for 1 h at 37°C in a humidified incubator under 5% CO_2_. After incubation, the medium was discarded, and the cells were washed with PBS, and then incubated with LPS (1 μg/mL, Sigma-Aldrich, MO, United States) for 3 h. The culture medium was replaced with basic DMEM before treatment. For the CON, the medium was replaced with basic DMEM incubated in a chamber 95% air 5% CO_2_. Finally, proteins were extracted for western blot analysis.

### Western Blotting Analysis for Tissues and BV-2 Cells

Mice of CON, CRS, RE + CRS groups were sacrificed, and then hippocampus derived from each group was frozen in liquid nitrogen and stored at -80°C. Tissue samples from various groups were homogenized with protein extraction reagent containing protease inhibitors. BV-2 cells of CON, LPS, and RE + LPS groups were lysed with lysis buffer, mechanically scraped off with a rubber scraper and centrifuged at 13,000 rpm for 25 min. Protein concentrations of both tissues and BV-2 cells were determined by a protein assay kit. Samples of supernatants containing 50 μg protein of tissues or 40 μg protein of BV-2 cells were loaded and heated to 95°C for 10 min, and were separated by sodium dodecyl sulfate poly-acrylamide gel electrophoresis in 10 or 12% gels, in a Mini-Protein II apparatus (Bio-Rad, CA, United States). Protein bands were electro-blotted onto polyvinylindene difluoride (PVDF) membrane and blocked with non-fat dried milk for 1 h. The membranes were incubated with BDNF (1:1000), TNF-α (1:1000), IL-1β (1:1000), p-NF κ B p65 (1:1000), Iba1(1:1000), p-AKT (1:1000), AKT (1:1000) and β-actin (1:3000) primary antibodies diluted in Tris-Buffered Saline-0.1% Tween (TBST) overnight at 4°C. After being washed with TBST three times, membranes were incubated with secondary antibodies, either with horseradish peroxidase conjugated anti-rabbit IgG (1:5000), for 1 h prior to being washed and developed with ECL reagents. The chemiluminescence signal was imaged using a ChemiDoc XRS system (Bio-Rad), and protein band signals were quantified by Image J 1.4.3.67 software. The signals of individual protein bands were normalized to the β-actin band intensity and represented in arbitrary units.

### Immunofluorescence Labeling in the Tissues and BV-2 Cells

Following deep anesthesia with 6% sodium pentobarbital, the mice were sacrificed by perfusion with 4% paraformaldehyde in 0.1 M PBS. The brain was removed and embedded in paraffin. Coronal sections of 7 μm thickness were cut on a microtome (Model: CUT5062; Mainz, SLEE, Germany). The sections were rinsed with PBS. For blocking of non-specific binding proteins, tissue sections were incubated in 5% normal goat serum diluted in PBS for 1 h at room temperature (22–24°C). After discarding the serum, the sections were incubated in a humidified chamber with primary antibody BDNF (1:100), TNF-α (1:100), IL-1β (1:100) and Iba1 (1:100) diluted with PBS overnight at 4°C. Following washing in PBS, sections were incubated, respectively, with fluorescent secondary antibodies: Cy3-conjugated secondary antibody (1:200) for 1 h at room temperature. After 3 rinses with PBS, the sections were mounted with a fluorescent mounting medium containing 4′, 6-diamidino-2-phenylindole, and were observed by inverted fluorescence microscope (Axio Observer. Z1, Zeiss, Germany).

BV-2 cells were fixed with 4% paraformaldehyde in 0.1 M PBS for 20 min. Following rinsing with PBS, the coverslips with adherent cells were used for immunofluorescence staining. In each group, BV-2 cells were incubated with the primary antibodies TNF-α (1:100), IL-1β (1:100) and NF κ B p65 (1:100) overnight at 4°C. Subsequently, the cells were incubated with fluorescent secondary antibodies: Cy3-conjugated secondary antibody and FITC conjugated lectin (1:200, *Lycopersicon esculentum*) that labels both microglia and blood vessel endothelial cells for 1 h at room temperature. After washing, the coverslips were mounted using a fluorescent mounting medium with 4′, 6-diamidino-2-phenylindole. The cells were observed by inverted fluorescence microscope.

### ELISA for IL-1β and TNF-α

ELISA kits were used for IL-1β and TNF-α determination in the supernatants. The kits were used following the recommendations of the manufacturer.

### Statistical Analysis

All data are presented as standard deviation or standard error of the mean. Group differences were assessed by one-way analysis of variance using SPSS 19.0. *P*-values < 0.05 were considered statistically significant.

## Results

### Antidepressant Effects of RE in CRS Mice

**FIGURE 1:**
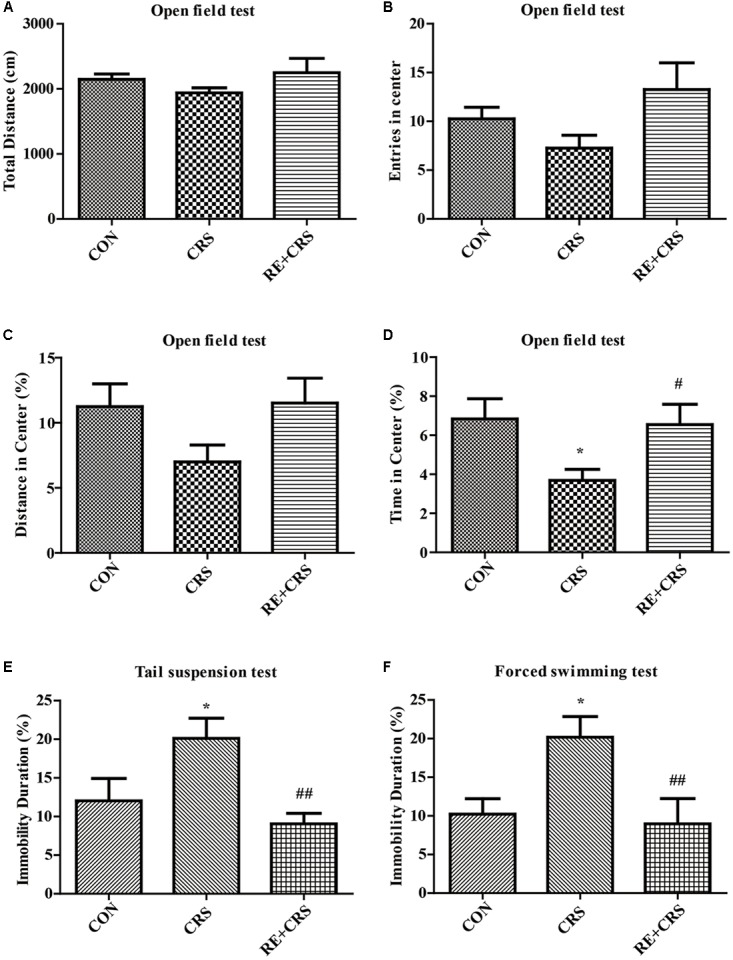
Antidepressant-like behaviors of RE in CRS mice. **(A)** Total distance in OFT. **(B)** Entries in center in OFT. **(C)** Distance in center in OFT. **(D)** Time in center in OFT. **(E)** Immobility duration in TST. **(F)** Immobility duration in FST. Data were shown as mean ± SEM. ^∗^*P* < 0.05, ^∗∗^*P* < 0.01 vs. CON; ^#^*P* < 0.05, ^##^*P* < 0.01 vs. CRS. *n* = 12 per group. CON, CRS and RE + CRS represent the control group, the group of chronic restraint stress, and the treatment group of rosemary extracts pretreatment plus chronic restraint stress, respectively.

To detect the antidepressant effects of RE, OFT, TST, and FST were examined in CRS model. Locomotor activity was evaluated by OFT, which showed no significant difference in total distance (*F*_2,33_= 1.451; *P* > 0.05; **Figure 1A**), entries in center (*F*_2,33_ = 2.537; *P* > 0.05; **Figure 1B**) and distance in center (*F*_2,33_ = 2.3; *P* > 0.05; **Figure 1C**). Time in center of OFT was significantly less in the CRS group than in the CON group, and this result was ameliorated by pretreatment with RE (*F*_2,33_ = 3.702; *P* < 0.05; **Figure 1D**), indicating that RE can protect CRS-induced anxiety. Immobility duration in TST (*F*_2,33_ = 5.789; *P* < 0.01; **Figure 1E**) and FST (*F*_2,33_ = 5.222; *P* < 0.05; **Figure 1F**) was enhanced in the CRS group than in the CON group. This phenomenon was reversed by pretreatment with RE. The descriptive characteristics are detailed in **Supplementary Table S1**. Altogether, these data indicate that RE play a vital role in antidepressant to CRS mice.

### RE Reversed Changes of Intestinal Microflora Induced by CRS

**FIGURE 2:**
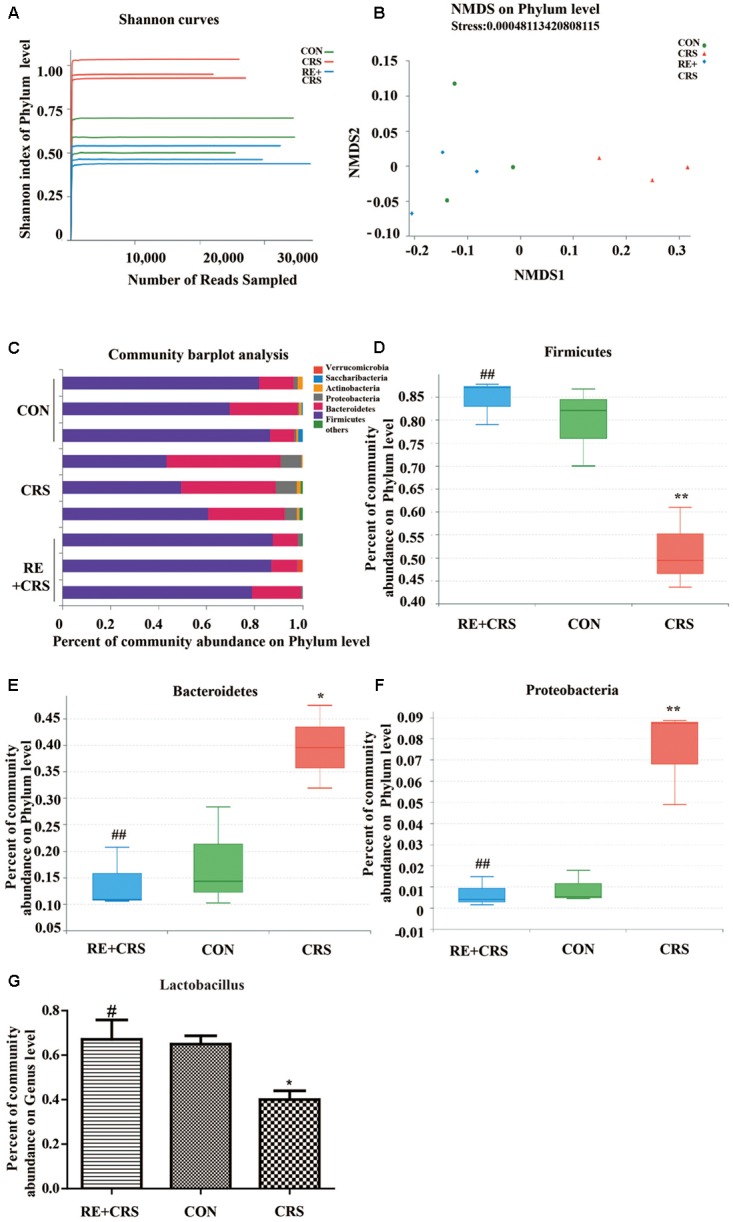
RE reversed changes of intestinal microflora induced by CRS. **(A)** Shannon curves on phylum level. **(B)** NMDS on phylum level. **(C)** Community barplot analysis on phylum level. One-way ANOVA box plot on phylum level about **(D)** Firmicutes, **(E)** Bacteroidetes and **(F)** Proteobacteria and on genus level about **(G)** Lactobacillus. Data were shown as mean ± SEM. ^∗^*P* < 0.05, ^∗∗^*P* < 0.01 vs. CON; ^#^*P* < 0.05, ^##^*P* < 0.01 vs. CRS. *n* = 3 per group. RE + CRS, CRS and CON represent the treatment group of rosemary extracts pretreatment plus chronic restraint stress, the group of chronic restraint stress, and the control group, respectively. The datasets of 16S rRNA sequencing for this study can be found in https://www.ncbi.nlm.nih.gov/sra/SRP158836.

Microbiota between mice exposed to chronic stress and non-stressed control mice show diverse composition (Bailey et al., 2011). To confirm the hypothesis that RE play a role in antidepressant to CRS mice related to rebalancing the gut microbiota, we directly measured the Shannon index, NMDS (non-metric multidimensional scaling), percent of community abundance, as well as proportion of sequences in Firmicutes, Bacteroidetes, Proteobacteria on phylum level and Lactobacillus on genus level. The results showed the Shannon index was enhanced in the CRS group than in the CON group, and the increased index was prevented by RE (**Figure 2A**), indicating the more species of microbiota in CRS group may result from an increased number of pathogenic bacteria. NMDS analysis was used to compare microbial community composition of each sample on phylum level. The result showed that microbial community composition in RE was more similar to CON as compared with CRS (**Figure 2B**). Community barplot analysis showed the community composition of intestinal microflora of three groups on phylum level (**Figure 2C**). To confirm that RE can reverse community composition of intestinal microflora of CRS, we further analyzed the proportion of sequences of Firmicutes, Bacteroidetes and Proteobacteria, which showed significant difference on phylum level by one-way ANOVA box plot. The abundance reduction of Firmicutes in CRS was reversed by RE (*F*_2,6_ = 16.287; *P* < 0.01; **Figure 2D**), While the sequences proportion enhancement of both Bacteroidetes and Proteobacteria was reversed by RE (*F*_2,6_ = 9.419; *P* < 0.05; *F*_2,6_ = 22.016; *P* < 0.01; respectively in **Figures 2E,F**). Additionally, RE reversed the abundance decline of Lactobacillus on genus level markedly **(***F*_2,6_ = 6.427; *P* < 0.05; **Figure 2G**). The descriptive characteristics are detailed in **Supplementary Table S2**. Taken together, these results demonstrate RE act a part in antidepressant to CRS mice related with rebalancing the gut microbiota.

### RE Increased BDNF as Well as p-AKT/AKT Expression and Reduced the Expression of Inflammatory Mediators in Hippocampus of CRS Mice

**FIGURE 3:**
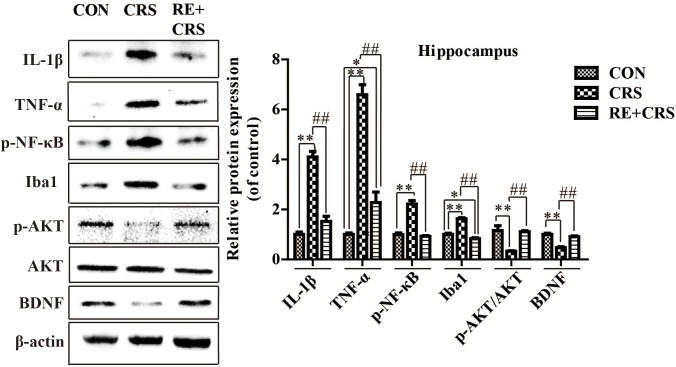
Protein expression of IL-1β, TNF-α, p-NF κ B, Iba1, p-AKT/AKT and BDNF in hippocampus. IL-1β, TNF-α, p-NF κ B, Iba1, p-AKT/AKT and BDNF protein expression in hippocampus was detected by western blot. Data were shown as mean ± SEM. ^∗^*P* < 0.05, ^∗∗^*P* < 0.01 vs. CON; ^#^*P* < 0.05, ^##^*P* < 0.01 vs. CRS. *n* = 4 per group. CON, CRS and RE + CRS represent the control group, the group of chronic restraint stress, and the treatment group of rosemary extracts pretreatment plus chronic restraint stress, respectively.

**FIGURE 4:**
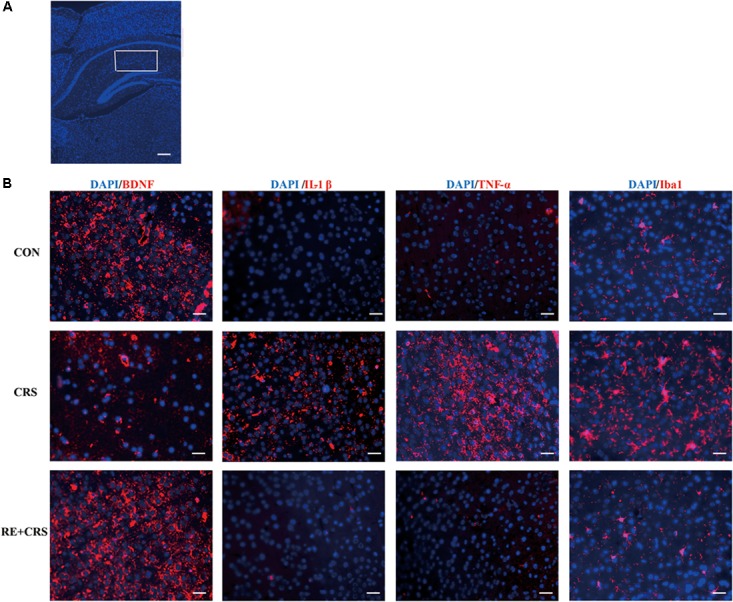
Effects of RE on protein expression of BDNF, IL-1β, TNF-α and Iba1 in hippocampus (Ca1 region). **(A)** Ca1 region was detected as shown by the box. Scale bars: 200 μm. **(B)** BDNF, IL-1β, TNF-α and Iba1 protein in hippocampus was detected by immunofluorescence, which was visualized by fluorescence microscopy. The images are representative of three experiments. Scale bars: 20 μm. CON, CRS and RE + CRS represent the control group, the group of chronic restraint stress, and the treatment group of rosemary extracts pretreatment plus chronic restraint stress, respectively.

To investigate whether there is a relationship between anti-inflammatory effects and antidepressant effects of RE, we chose the depression and inflammation related brain area, hippocampus, to examine expression of inflammatory cytokines under CRS stresses. Western blot analysis showed that the protein expression of IL-1β, TNF-α, p-NF κ B p65 and Iba1 in hippocampus was obviously elevated by CRS as compared with CON, and suppressed by RE compared with CRS mice (*F*_2,9_ = 81.089; *F*_2,9_ = 76.164; *F*_2,9_ = 64.568; *F*_2,9_ = 90.507; respectively; *P* < 0.01; **Figure 3**). It was striking that BDNF and p-AKT/AKT expression in CRS mice was less than CON, and RE group showed the most drastic increase as compared with CRS group (*F*_2,9_ = 43.556; *F*_2,9_ = 16.523; respectively; *P* < 0.01; **Figure 3**). The descriptive characteristics are detailed in **Supplementary Table S3**. Protein expression of BDNF, IL-1β, TNF-α and Iba1 in hippocampus (Ca1 region) was verified by immunofluorescence (**Figure 4**). These results suggest that RE can increase protein expression of BDNF as well as p-AKT/AKT and reduce inflammatory mediator proteins’ expression to act antidepressant function. Additionally, the antidepressant functions of RE maybe related with its anti-inflammatory effects and regulation of BDNF as well as p-AKT/AKT.

### RE Reduced the Expression of Inflammatory Cytokines in LPS-Induced BV-2 Microglia

**FIGURE 5:**
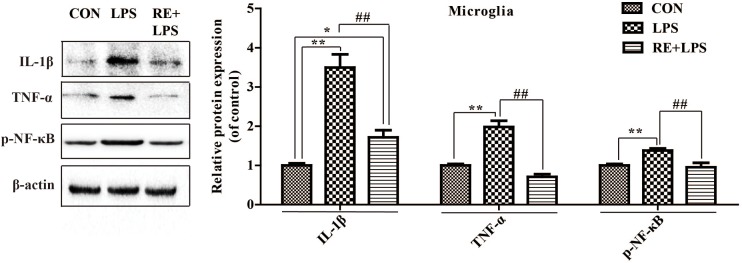
Protein expression of IL-1β, TNF-α, and p-NF-κ B p65 in microglia pretreated by RE. BV-2 microglia were incubated with RE (5 μM, 5 μM was the amount of substance of carnosic acid, and RE contained 60% carnosic acid.) for 1 h, prior to treatment with LPS for 3 h. Western blot analysis was performed to examine IL-1β, TNF-α, and p-NF-κ B p65 expression. Data were shown as mean ± SEM. ^∗^*P* < 0.05, ^∗∗^*P* < 0.01 vs. CON; ^#^*P* < 0.05, ^##^*P* < 0.01 vs. CRS. *n* = 4 per group. CON, LPS and RE + LPS represent the control group, the LPS group, and the treatment group of rosemary extracts pretreatment plus LPS, respectively.

**FIGURE 6:**
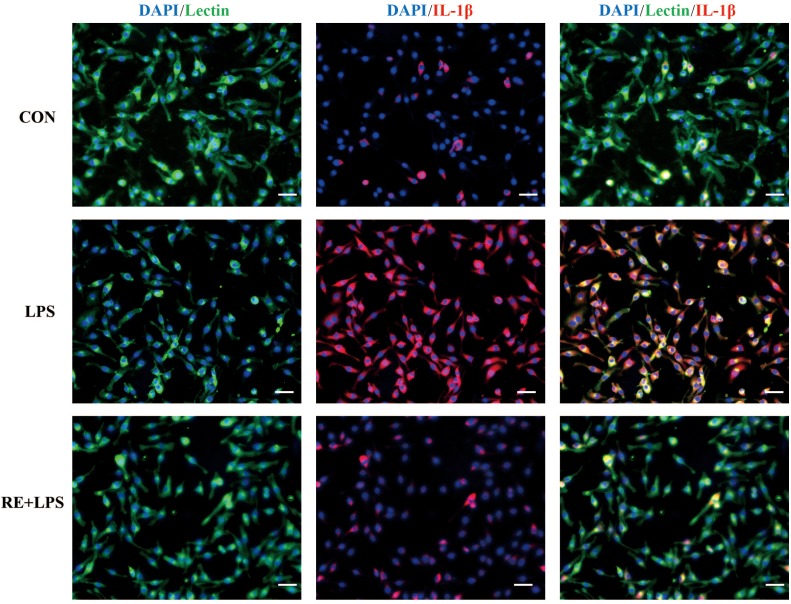
Reduction of IL-1β expression in activated microglia pretreated by RE. BV-2 microglia were incubated with RE (5 μM, 5 μM was the amount of substance of carnosic acid, and RE contained 60% carnosic acid.) for 1 h, prior to treatment with LPS for 3 h. IL-1β protein was detected by immunofluorescence, which was visualized by fluorescence microscopy. Scale bars: 50 μm. CON, LPS and RE + LPS represent the control group, the LPS group, and the treatment group of rosemary extracts pretreatment plus LPS, respectively.

**FIGURE 7:**
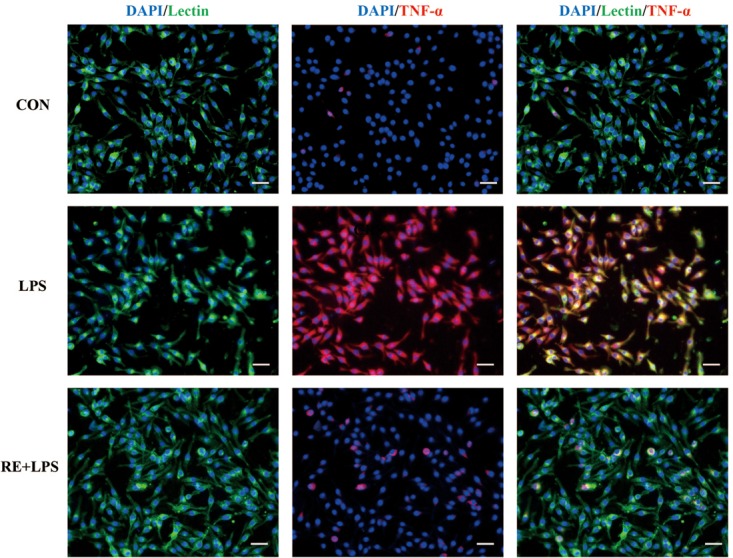
Reduction of TNF-α expression in activated microglia pretreated by RE. BV-2 microglia were incubated with RE (5 μM, 5 μM was the amount of substance of carnosic acid, and RE contained 60% carnosic acid.) for 1 h, prior to treatment with LPS for 3 h. TNF-α protein was detected by immunofluorescence, which was visualized by fluorescence microscopy. Scale bars: 50 μm. CON, LPS and RE + LPS represent the control group, the LPS group, and the treatment group of rosemary extracts pretreatment plus LPS, respectively.

**FIGURE 8:**
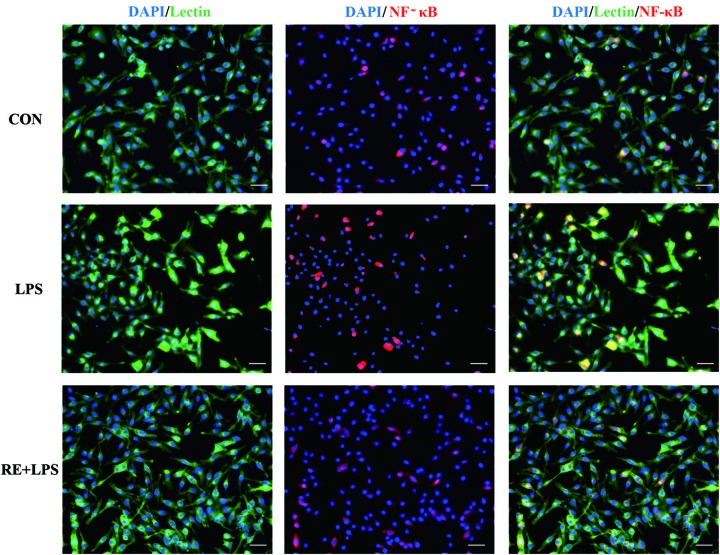
Reduction of NF-κ B expression in activated microglia pretreated by RE. BV-2 microglia were incubated with RE (5 μM, 5 μM was the amount of substance of carnosic acid, and RE contained 60% carnosic acid.) for 1 h, prior to treatment with LPS for 3 h. NF-κ B protein was detected by immunofluorescence, which was visualized by fluorescence microscopy. Scale bars: 50 μm. CON, LPS and RE + LPS represent the control group, the LPS group, and the treatment group of rosemary extracts pretreatment plus LPS, respectively.

Western blot analysis and immunofluorescence results showed that changes in the inflammatory mediators, including TNF-α, IL-1β and NF-κ B, were also observed in LPS-induced BV-2 cells to be consistent with results *in vivo*. IL-1β, TNF-α and NF-κ B protein expression (*F*_2,9_ = 33.066; *F*_2,9_ = 44.27; *F*_2,9_ = 9.406; respectively; *P* < 0.01; **Figure 5**) and immunofluorescence intensity (**Figures 6–8**) were vitally augmented vs. CON when the cells were subjected to LPS, but were clearly diminished when pretreated with RE. The descriptive characteristics are detailed in **Supplementary Table S4**. These results indicate that RE can alleviate inflammatory mediator proteins’ expression to protect BV-2 microglia cells activated by LPS.

### RE Reduced IL-1β and TNF-α Levels Augmented by CRS in Mice Serum

**FIGURE 9:**
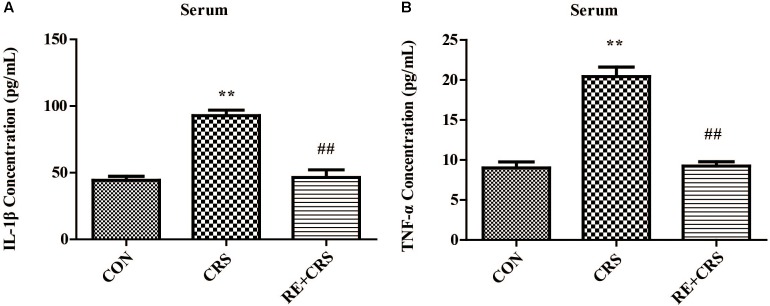
RE reduced IL-1β and TNF-α levels augmented by CRS in mice serum. CRS Mice were pretreated with RE (100 mg/kg) for 21 days. **(A)** IL-1β and **(B)** TNF-α levels in serum were detected by ELISA. Data were shown as mean ± SEM. ^∗^*P* < 0.05, ^∗∗^*P* < 0.01 vs. CON; ^#^*P* < 0.05, ^##^*P* < 0.01 vs. CRS. *n* = 3 per group. CON, CRS and RE + CRS represent the control group, the group of chronic restraint stress, and the treatment group of rosemary extracts pretreatment plus chronic restraint stress, respectively.

To confirm the anti-inflammatory effects of RE, we detected the mice serum of three groups by using ELISA. IL-1β and TNF-α levels were markedly enhanced in CRS mice serum compared with CON, and significantly lessened in RE as compared with CRS (*F*_2,6_ = 37.362; *F*_2,6_ = 55.861; respectively; *P* < 0.01; **Figure 9**). The descriptive characteristics are detailed in **Supplementary Table S5**. Taken together, these results suggest RE play a peripheral anti-inflammatory role to act antidepressant function.

## Discussion

Increased systemic inflammation reactions and serum inflammatory biomarkers are reported to be the pathogenesis of most MDD, and the features of poor prognosis for MDD (Schmidt et al., 2011). It is believed that bacteria, involved in key processes to maintain body homeostasis and populate the human gut, can modulate inflammation and brain functions, including mood and behavior (Alam et al., 2017; Pisanu and Squassina, 2017). RE have various pharmacological effects, including anti-proliferative activity on colon cancer cells (Sanchez-Camargo et al., 2016), anti-inflammatory activity in primary macrophages (Justo et al., 2015), protecting from ultraviolet harmful effects in skin (Perez-Sanchez et al., 2014), and inhibitory effects on the growth of various human cancer cell lines (Yesil-Celiktas et al., 2010). In the present study, we obtained the first evidence that RE acted a role of antidepressant against CRS-induced depressive-like behaviors as well as increased hippocampal and serous inflammatory cytokines in mice, and we further explored the gut microbiota changes facilitating these antidepressant effects. Additionally, we investigated the protective function of RE against LPS-induced activation of BV-2 microglia.

Considerable evidence indicates that CRS contributes to depression (Chiba et al., 2012; Xu et al., 2017), via activation of inflammatory pathways (Norman et al., 2010; Aalling et al., 2018), due to the microbiota-gut-brain axis (Liang et al., 2015), and the model of CRS is widely used to mimic the effects of inflammation induced depression (Norman et al., 2010; Guo et al., 2012; Aalling et al., 2018). Moreover, LPS induces pro-inflammatory responses in BV-2 microglia and thus leads to microglia-mediated neuronal damage (Qin et al., 2018). We found depressive-like behaviors, increased inflammatory cytokines in serum and hippocampus, and gut microbiota dysbiosis in CRS mice, as well as upregulation of inflammatory cytokines in microglia activated by LPS. Our results showed that pretreatment with RE improved depressive-like behaviors, attenuated inflammatory cytokines and rebalanced gut microbiota in CRS mice. Moreover, pre-incubation with RE protected microglia by alleviating inflammatory cytokines induced by LPS.

Antidepressant drugs present antimicrobial effects related to the effectiveness for MDD, which is associated with changes in gut permeability and microbiota composition (Macedo et al., 2017). Additionally, the changes result in an increase in both the diversity and richness of gut bacterial populations in patients with MDD (Jiang et al., 2015). The depression-like behavior induced by prolonged high-fat diet in mice is associated with distinct alterations of intestinal microbiome including diminished abundance of Bacteroidetes and increased abundance of Firmicutes and Cyanobacteria (Hassan et al., 2018). Glyphosate-based herbicides induce an increase of anxiety and depression-like behaviors of mice, and additionally herbicides decrease the relative abundance of Corynebacterium, Firmicutes, Bacteroidetes and Lactobacillus in gut (Aitbali et al., 2018). Bacteroidetes, Proteobacteria, and Actinobacteria increase, but Firmicutes reduces in the major depressive disorder groups compared with the healthy control group (Jiang et al., 2015). Women who receive Lactobacillus rhamnosus HN001 have significantly lower depression and anxiety scores in the postpartum period (Slykerman et al., 2017). Behavioral, cognitive, and biochemical aberrations of rats caused by CRS depression model are improved by *L. helveticus* NS8 (Liang et al., 2015). These data reinforce the essential link between the depression and abundance of Lactobacillus, Bacteroidetes, Firmicutes, and Proteobacteria. RE modifies microbiota composition and decreases β-glucosidase activity in the caecum of female Zucker rats (Romo-Vaquero et al., 2014), and it also owns antidepressant and anxiolytic activities in mice (Abdelhalim et al., 2015). Accordant with previous reports, our data demonstrated that RE could decrease the diversity of gut microbiota, lessen the sequences proportion of both Bacteroidetes and Proteobacteria, and promote abundance of Lactobacillus and Firmicutes, which suggest the antidepressant effects of RE stemmed from rebalance of gut microbiota.

Depression derives from activation of immune-inflammatory pathways, according to that the release of IL-1β and TNF-α triggers off neuroendocrine and neurochemical deterioration (Dowlati et al., 2010; Shelton et al., 2011; Hiles et al., 2012; Zhao et al., 2017). Consistent with the effect of ketamine (Tan et al., 2017), RE acted a part in anti-depressive effects on TST and FST by suppressed the level of TNF-α and IL-1β in hippocampus, serum and microglia. TNF-α can activate NF-κB signaling pathway in endothelial cells (Zhou et al., 2017), which is involved in regulation of adult neurogenesis and in effects of drugs that are endowed with antidepressant activity (Bortolotto et al., 2014). Our study showed RE relieved NF- κ B level in hippocampus and microglia. There is interaction between cortical spreading depression and Iba1 immunoreactivity in the rat motor cortex (Lima et al., 2014). RE eased Iba1 expression in hippocampus induced by CRS in our data. Depressed patients have lower BDNF levels compared with non-depressed subjects (Monteiro et al., 2017; Youssef et al., 2018); therefore, one of therapeutic strategy for treating depression is to upregulate the BDNF expression (Khan et al., 2018). RE increased BDNF expression in hippocampus alleviated by CRS, demonstrating the antidepressant efficiency of RE. Accumulating evidences suggest sirtuins 6 is involved in the modulation of depressive-like behaviors and affects the survival and synaptic plasticity of hippocampal neuron via decreasing the ratio of p-AKT/AKT in the primary hippocampus neurons (Mao et al., 2017). In our results, RE played a part in antidepressant by elevating p-AKT/AKT expression in hippocampus. Depression is due to the increased production of pro-inflammatory cytokines by microglia activation, and fluoxetine can attenuate LPS-induced production of IL-1β and p-NF-κB expression in microglia (Yang et al., 2014). In accordance with the previous study, our results showed that RE alleviated IL-1β, TNF-α and p-NF-κB expression in BV-2 cells induced by LPS, indicating the protective effect to BV-2 cells of RE.

Taken together, the results of our research clearly manifest that the antidepressant effects of RE are mediated by anti-inflammatory effects in hippocampus, serum and BV-2 cells as well as rebalance of gut microbiota.

## Conclusion

**FIGURE 10:**
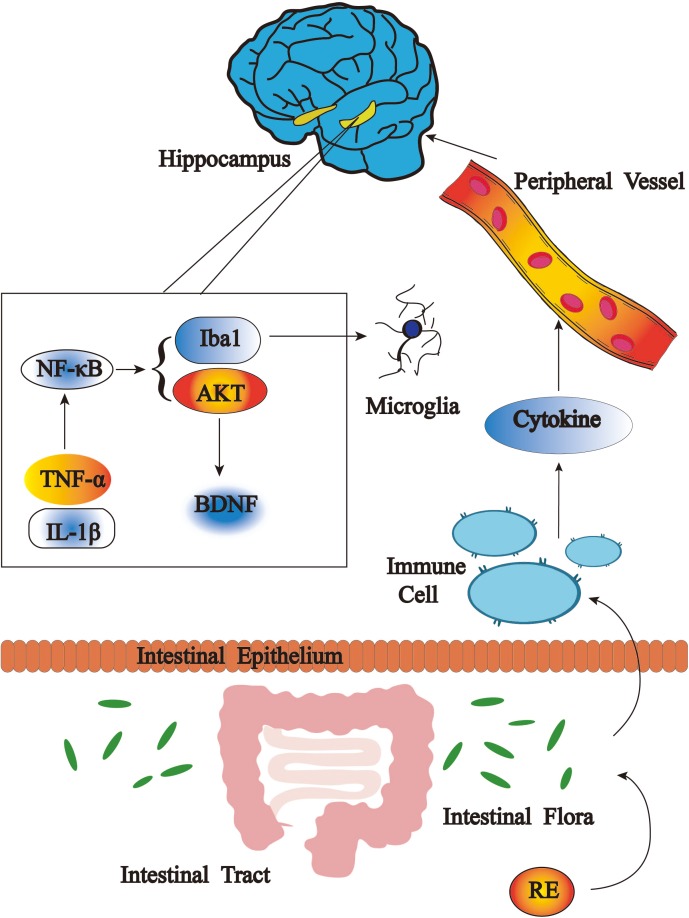
Antidepressant effects of RE as related to anti-inflammatory effect and rebalance of gut microbiota.

Our results represent the first evidence that RE significantly ameliorates depressive-like behaviors in CRS mice. This effect was associated with the inactivation of inflammatory reactions in hippocampus, serum as well as BV-2 cells and the promotion of BDNF and p-AKT/AKT expression in hippocampus, as well as rebalance of gut microbiota (**Figure 10**). Our data will provide more insights into inflammation, gut dysbiosis and microglia as essential targeting therapies for depression.

## Author Contributions

YG, JX, RZ, and HY contributed to the conception of the study. HL, HY, and LZ contributed significantly to analysis and manuscript preparation. YG, XL, WH, and YY performed the data analyses and wrote the manuscript. RZ and JX helped to perform the analysis with constructive discussions.

## Conflict of Interest Statement

The authors declare that the research was conducted in the absence of any commercial or financial relationships that could be construed as a potential conflict of interest.

## Footnotes

1 http://drive5.com/uparse/

2 http://rdp.cme.msu.edu/

3 http://www.i-sanger.com
